# Porcine Circovirus Type 2 Pathogenicity Alters Host’s Central Tolerance for Propagation

**DOI:** 10.3390/pathogens9100839

**Published:** 2020-10-13

**Authors:** Xaver Sidler, Titus Sydler, José Maria Mateos, Stefanie Klausmann, Enrico Brugnera

**Affiliations:** 1 Department of Farm Animals, Division of Swine Medicine, Vetsuisse Faculty, University of Zurich, 8057 Zurich, Switzerland; stef.klausmann@gmx.de; 2 Institute of Veterinary Pathology, Vetsuisse Faculty, University of Zurich, 8057 Zurich, Switzerland; tsyd@vetpath.uzh.ch; 3 Center for Microscopy and Image Analysis, University of Zurich, 8057 Zurich, Switzerland; jose.mateos@zmb.uzh.ch

**Keywords:** porcine circovirus type 2, PCV2d, reproductive organs of stillborn fetuses, embryos, gyrovirus, central tolerance

## Abstract

Porcine circovirus type 2 (PCV2) infections and resulting diseases are a worldwide threat to pig production. PCV2 bears a uniqueness that allows for us to understand more about chronic infections and the immune system in general. The virus can be phylogenetically subdivided into PCV2a to PCV2h genotypes. Although vaccination against PCV2 has been seen to prevent the manifestation of PCV disease, PCV2 still lingers as subclinical infections in all developmental stages of pigs. The “slow and low” tactic gives PCV2 a particular advantage in a host’s immune surveillance. Since the inception of the PCV2 associated panzootic, research scientists have been trying to understand the pathogenicity of PCV2. Different research groups found that one genotype group member was more pathogenic than others. We found, in our weaner infection model with in vivo transfection of different recombinant PCV2 genotype group members that these viruses alter T cell maturation in the thymus, including host’s central tolerance. Here, we extend these original observations by showing that PCV2 infected cells were also found in proximity within the female and male reproductive organs of stillborn pig fetuses. These PCV2 pools were sufficient in infecting three and half-day-old embryos in sows. Furthermore, the dominant PCV2 group member was more pathogenic in our weaner infection model. PCV2 pre-immunocompetence infection makes PCV2 recognized by central immune tolerance as belonging to the host. This also explains why pathogenicity is not a genetically intrinsic characteristic of PCV2; however, the dominance of any one PCV2 genotype group member leads to a more efficient deletion of the T cells against that specific genotype group member in the thymus.

## 1. Introduction

With 1766–1768 nucleotides, porcine circovirus type 2 (PCV2) is the smallest genomic panzootic associated virus among the circular single-stranded DNA (ssDNA) viruses hitherto. These ssDNA viruses are remarkably understudied, and only recently have researchers begun to study these viruses due to the advent of next-generation sequencing in birds, fish, mammals, and even humans [1,2].

The small genome of PCV2 encodes at least nine different transcripts [3]. Among them, open reading frame 1 (ORF1) codes for the replication-associated protein (Rep) and ORF2 encodes the viral capsid protein (Cap) that is used as the standard for the phylogenetic analysis of the PCV2 genotype group members [4]. The PCV2 genome is ambisense, i.e., the encapsidated single-stranded viral DNA strand serves as a plus strand for the *rep* gene and as a minus strand for the *cap* gene [4].

The success of the virus in pigs might also be attributed to its high genomic plasticity [5]. Because the virus genome is single-stranded and replicates through a rolling circle mechanism, it is prone to mutation [6], and genotype group member co-replication [7] increases virus variability by recombination [7,8], thereby providing the virus an advantage to escape elimination by the host’s immune system. This resulted in several hundred different virus genotype group member sequences that were observed and deposited in the National Center for Biotechnology Information (NCBI) databases [9,10]. These PCV2 nucleotide sequences can be classified into eight major genotypes by basically using the viral Cap sequence for categorization: PCV2a to PCV2h [9,10]. Among these genotypes was also the PCV2d genotype group member, which was proposed as more virulent and pathogenic than the classical PCV2a or PCV2b genotype group members [11]. This genotype group member was later identified as the genome with the NCBI accession number JX512856 [12] that we had cloned and used in our weaner cotransfection experiments [13].

Many research scientists in the field overlook the fact that *Circoviridae* family members of PCV2 are also associated with the so-called “slow and low” tactic [14] i.e., these family members are constantly replicating at low levels [15,16] to not disturb the host’s delicate energy balance or cause major damage to the host’s tissue. This way, they lay low not to inadvertently trigger the host’s immune response. Thus, the *Circoviridae* family can hardly be differentiated from latent viruses [14]. This latent nature explains PCV2s’ low virulence and the need for a cofactor to induce disease symptoms in a healthy host [13,17,18].

Among the many *Circoviridae* family members, PCV2 claimed awareness, as it was the primary pathogen that was responsible for clinically distinct diseases and panzootics in pigs, named composite PCV2 diseases (PCVD) [16]. With pig vaccination working against the clinical manifestation of PCV2, PCVD lost their grip on pig production. Vaccination efficiently reduced the impact of PCVD; it lowered the cull and mortality rates and significantly promoted improved average daily weight gains [19,20,21]. Although vaccination became an integral part of good pig husbandry, the virus continued to linger in pig farms or individuals [22,23,24]. Several hypotheses might, on their own or together, explain the continuous viral presence, e.g., the hallmark of the *Circoviridae* family is its surprisingly high viral resistance to adverse conditions [25], and this unremittingly presents a virus pool for new infections. Many researchers suggest that the majority of infections occur through horizontal infections [22,26] (e.g., through the introduction of a newly infected pig into the pre-existing herd). Thus, horizontal transmission of PCV2 is more important than vertical transmission to its pathogenicity [22,26]. However, we favor the idea that PCV2 infections first occur early in pigs’ fetal ontogeny [24], and this underlying infection prepares individuals as a viral conducive PCV2 “incubator” through altered host’s central tolerance [13]. We have also shown peripheral T cell anergy in PCVD with high PCV2 antigen concentrations [13]. Nevertheless, what makes PCV2 pathogenicity so special is that it uses thymocyte negative selection for its propagation [13], as seen in the almost inexistent T cell response to the PCV2 Cap protein [27]. The process of central tolerance is confined to the host’s bone marrow and thymus [28], and it also indicates the antigens’ presence before immunocompetence [28,29]. Central tolerance in the thymus is the process of avoiding the production of self-reactive T cells. In other words, it is basically the deletion of thymocytes with T cell receptors (TCR) that have a high affinity to the host’s own tissue. This is also called thymocyte negative selection [28]. To this end, we presented evidence from different perspectives of the PCV2 pathogen over the last years, starting with the original manuscript of Klausmann et al. [13]. We showed the presence of latent PCV2 infected cells in the thymus [13,24]; the PCV2 antigen presenting cells at the corticomedullary junction that led to the deletion of the maturing thymocytes that are predominant in the medulla of the thymus during PCVD [13]; the presence of latent infected cells at the corticomedullary junction and medulla; and, the downregulation of TCR and concomitant upregulation of CD4-coreceptors [13]. Furthermore, all of the fetuses were infected with PCV2 in the thymus independently of anti-PCV2 vaccination, even before immunocompetence [24]. The irrational anti-PCV2 IgG expression would also be a clue of an unusual T cell response [24,30] and possibly the early adaption of PCV2 to a new host, cattle [31].

What follows is additional supporting results and information of how the PCV2 genotype group members become part of the hosts’ central tolerance and thereby giving the impression that one PCV2 genotype group member may be more pathogenic than another: (a) we show vertical transmission in early embryo development through PCV2 presence within and in proximity to both female and male reproductive organs, and, PCV2 infections already present in embryos as early as 3–4 days old before immunocompetence, and (b) four different experimental settings indicate that, in general, one PCV2 genotype group member is not more pathogenic when compared to another and, instead, the dominant infection of the PCV2 genotype group member in the pig cohort and the individuum determines the PCV2 genotype group member pathogenicity. This, we show by sequence analysis of stillborn fetuses and 3–4 days old embryos, by comparing the virulence of PCV2a to PCV2b/PCV2d (PCV2b/d) genotype group members in the in vivo weaner transfection model and by sequences of a competition experiment with weaners in vivo transfected with PCV2a/PCV2b/PCV2d (PCV2ab/d) genotype group members [13].

## 2. Results

### 2.1. Stillborn Fetuses Infected with PCV2b Genotype Group Members in Female and Male Genital Tract and Associated Lymph Nodes

**Figure 1:**
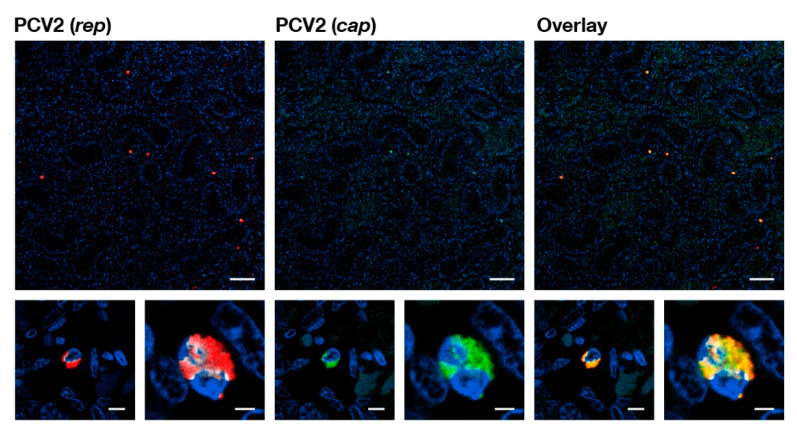
Confocal microscopy of a fluorescence in situ hybridization (FISH) stained paraffin-embedded testis tissue section of a high PCV2 “latent” infected (+++) stillborn fetus. The panels show, from left to right, the fluorescent hybridization signals of the PCV2 oligonucleotide recognizing *rep* (red), the oligonucleotide for PCV2 oligonucleotide recognizing *cap* (green), and the overlay of the signals (yellow). The nuclei (DAPI stained) appear blue. The lower panels show two different magnifications of two different places at the top panel. The white bar indicates the reference length of 50 μm for the top panels and the reference lengths of 5 μm and 2 μm alternates for the lower panels.

We investigated the sexual tracts in 31 male and 24 female stillborn fetuses (see Table 1). Thirty-three stillborn fetuses that were randomly investigated carried PCV2 infected cells in the thymus by fluorescence in situ hybridization (FISH) analysis. None of the 55 stillborn fetuses contained detectable PCV2 capsid proteins by histochemistry. We found various grades of “latent” PCV2 infections in both male and female reproductive tracts, although the male reproductive tract, in general, contained more PCV2 infected cells by FISH analysis. We also noticed that 21 of the 31 males’ testicles contained PCV2 infected cells by FISH analysis (Figure 1). Nine of these 21 males also contained PCV2 infections in the epididymis, with fewer PCV2 infected cells here than in the testicles. We also found that non-epithelial cells of the accessory sexual glands tested positive for PCV2 infected cells by FISH analysis. The investigated females, in general, contained, per tissue section, only 1–5 infected cells, as visualized by FISH analysis; these infected cells were mostly in the ovaries, followed by the oviduct, and the uterus horn. The ovaries of 18 of the 24 females analyzed contained PCV2 infected cells.

**Figure 2:**
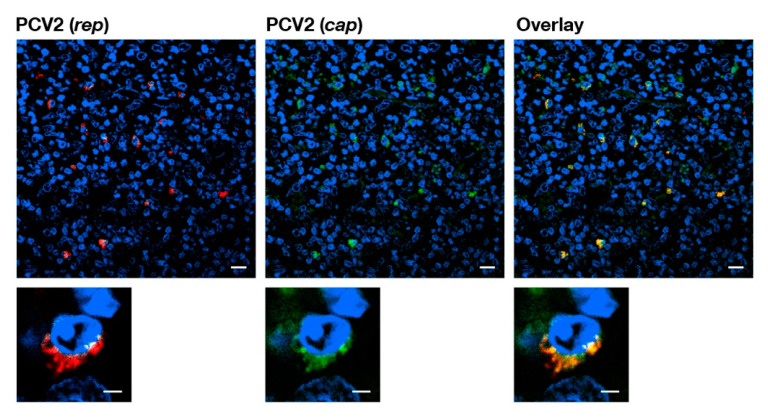
Confocal microscopy of a FISH stained paraffin-embedded sexual tract associated lymph node (+++) of a female stillborn fetus. The panels show, from left to right, the fluorescent hybridization signals of the PCV2 oligonucleotide recognizing *rep* (red), the oligonucleotide for PCV2 oligonucleotide recognizing *cap* (green), and the overlay of the signals (yellow). The nuclei (DAPI stained) appear blue. The lower panel shows a different magnification of the top panel. The white bar indicates a reference length of 10 μm for the top panels and a reference length of 2 μm for the lower panels.

We only found interstitial cells of both sexual tracts to be infected with PCV2. We were unable to find any evidence that the gametes were directly PCV2 infected in male or female stillborn reproductive tracts by FISH analysis. Furthermore, all of the identified genital tract associated lymph nodes from 18 stillborn fetuses of both sexes were carrying many PCV2 infected cells (Figure 2).

Furthermore, we were able to identify, from eight females (about 33%) and five males (about 16%), the dominant PCV2 genotype infection of the reproductive tracts by sequencing the amplified nested polymerase chain reaction (PCR)-product of 180 base pairs, which overlaps the so-called PCV2 signature motif: the dominant PCV2 genotype group member was of the PCV2b genotype (Table 1, Table S1), similar to JX512857.

### 2.2. Embryos Are Naturally Infected with PCV2b Genotype Group Members

We collected embryos from three sows immediately after slaughter and three and a half days after the first insemination in order to determine at what earliest stage of embryo development can PCV2 infection be detected. The embryos from the first sow were collected in four different tubes, all of these four samples were found to be infected with the PCV2b genotype group member/s, which was/ were similar to JX512857 according to the amplified PCR product by sequencing. The elution fluid was also collected in four different samples after rinsing the oviduct, of which three were also identified as carrying a PCV2 genotype group member similar to JX512857 by sequencing. From the second sow, three tubes of embryos were collected and two were positive for PCV2b group member/s, similar to JX512857, which was/were also found in the embryos of sow one. The two elution fluids did not give any amplificate. From the third sow, two tubes of embryos were collected, of which we received no PCR amplificate, and the two elution fluids were also void of any PCV2 sequences.

### 2.3. Determining Whether PCV2a or PCV2b/d Group Members Are More Virulent in Weaners

In previously published study, we mimicked PCV2 pathogenicity by transfecting recombinant PCV2 genotype group members in the presence of cyclosporine A immunosuppressant in weaners. We separately in vivo transfected two additional groups, each consisting of four male weaners, with one group in vivo transfected with PCV2a genotype group members and the second group with PCV2b/PCV2d genotype group members, which we collectively termed PCV2a- or PCV2b/d-weaners, respectively.

In both PCV2a and PCV2b/d groups (see Table 2 and Table 3), we observed that the infection was successful as each pig individually mounted an anti-PCV2 IgM response to the injected recombinant PCV2a or PCV2b/d genotype group members between the 20-day post-transfection (p.t.) and 41-day p.t. period. Only two pigs of the PCV2b/d-weaners had a remaining measurable IgM-B cell response at day 51 p.t (Table 3). No meaningful anti-PCV2 IgG responses were observed in any of the transfected pigs over the 51-day period (Table 3). Although all four pigs of the PCV2b/d-weaners exhibited a higher than 10^6^ PCV2-templates per ml (Table 2 and Table 3), we only found one of the four PCV2a-weaners exhibit a measurable viremia over 10^5^ PCV2-templates per ml once over the whole period of 51 days p.t. In one weaner, we saw centrofollicular PCV2 antigen positive cells by immunohistochemistry (IHC) in the tonsil of a PCV2b/d transfected weaner (Table 2). None of the pigs developed PCVD (Table 2).

### 2.4. Genotype Group Member Competition and Efficiency in the Weaner PCV2 Transfection Model

From a separate, previous weaner transfection experiment, we analyzed 20 blood samples that were transfected with a minimal concentration of PCV2a, PCV2b, and PCV2d genotype group member DNA mix. It took at least 13 days p.t. until any relevant PCV2 concentrations were measured in the blood. In this setting, concurrent genotype group members in the same pig allowed for the testing of the evolution of PCV2 infection in order to determine which of the genotype group member/s prevailed and were more virulent in direct competition. To this end, we analyzed the complete ORF2 of PCV2 by PCR and sequencing from the in vivo transfected pigs. We analyzed eight blood samples from eight weaners belonging to the so-called PCV2ab/d-weaners that were previously transfected with PCV2a, PCV2b, and PCV2d genotypes group members. We also analyzed nine blood samples from seven weaners of the PCV2ab/d & CysA-weaners, which originated from weaners that were also previously transfected with the three genotype group members and additionally treated with cyclosporine A. Two of these nine blood samples included two additional different time points. We further analyzed two blood samples from uninfected and untreated so-called control weaners and one blood sample from a so-called CysA-weaner that was only treated with cyclosporine A. These three weaners’ blood samples gave us indications regarding the dominant PCV2 genotype group member and the PCV2 background infection of the cohort. For whole PCV2 ORF2 amplification, we chose the highest measured PCV2 concentration during the 51-day p.t., except for two PCV2ab/d & CysA weaners, of which the data set was extended by two additional time points. This way, we analyzed nine different blood samples of the Institute of Virology and Immunology (IVI, Mittelhäusern, Switzerland) weaner cohort, and eleven from the weaner cohort at the University of Zurich. Eighteen of the twenty whole PCV2 ORF2 amplificates were identical with the PCV2b genotype group member, JX512857, by sequencing. Two weaners of four PCV2ab/d & CysA groups from the IVI were identified as different by sequencing: one carried a complex mix of PCV2 ORF2 populations while the other carried a mix of JX512857 and either JX512858 or JX512860, or both.

## 3. Discussion

The current presented data provide further evidence that the major pathogenicity of PCV2 is to become part of the host before the adaptive responses’ development [24] and, thus, subverting the host’s central tolerance [13]. Previously, we found the presence of PCV2 before immunocompetence in the pig [24]; PCV2 interference with thymocyte maturation at their positive and negative selection stage [13]; and, the generally ascribed lymphatic trophic PCV2 nature [22]. In this report, we specifically show that the multicellular development stages of pig embryos already carry PCV2, even before settlement in the uterus. Even though the gametes in the female and male fetuses were not directly infected, we discovered that a constant PCV2 infection pool was provided from accessory cells and/or adjunct lymph nodes (Table 1, Figure 2) of both the female and male reproductive tracts of stillborn fetuses (Table 1, Figure 1) for the first time. We think that our study extends and complements the previous observations of Bielanski et al. [32] that 74% of gilts carry PCV2 infections in the reproductive tract: we analyzed fetuses’ reproductive tracts of both pig genders before environmental PCV2 contamination perturbed the data as it had most likely occurred in gilts [32]. In our study, stillborn sexual tracts were the best choice to further understand PCV2 vertical transmission, as they had the highest likelihood of finding PCV2 infections via vertical transmission without the interference of exogenous PCV2 infections, including other reasons, such as the visibility of the reproductive tract segments and 3R conformity.

Additionally, the literature supports that 22–47% of the boar ejaculate was contaminated with PCV2 [33,34,35,36,37]. These are probably small percentages of PCV2 when compared to the actual situation when one takes into account the dilution factor and the general difficulties in amplifying DNA from ejaculate, and the fact that boars have an even greater cellular density when compared to cattle [38,39]. From these data and our data, one would expect high infection prevalence of the pig fetuses. However, this was not observed at first, as less than 1% of the pig fetuses were reported as carry PCV2 at the beginning of the millennium [40]. Nevertheless, these data were observed with pooled fetal tissues as source material for PCR amplification and, thus, the dilution factor should not be underestimated. Other questions also arose regarding the sensitivity of the used PCR reaction. From our experience with optimized PCR reaction on recombinant template amounts, we missed out on PCV2 template numbers smaller than 100 templates/µL DNA eluate and, thus, it is quite easy to imagine that positive samples would be indicated as negative for PCV2 depending on the sensitivity of applied PCR reaction. Other researchers found that PCV2 spiked ejaculate could not only infect, but also provoke, reproductive failure [41,42]. The question that needs to be posed is whether the cell culture propagated virus samples were contaminated with active substances (e.g., cytokines, viruses that keep the cell line immortal, etc.), which might have interfered with the pregnancy of the investigated sows. It was also noticed that small amounts of PCV2 in boar ejaculate were not uncommon [33,36,37]. Moreover, pig embryos without zona pelucida were 100% infected with PCV2 in vitro and, more importantly, the viability of the infected embryos was unaffected [43]. These findings support that most of the embryos would be unaffected by a small PCV2 infection in the earliest moments of their development. This is also supported by our previous [24] and present observations. Therefore, the probability is high that this early PCV2 infection that we report in the present manuscript also occurred commonly in the field under natural conditions as both male and female sexual tracts carried PCV2 infected cells. These numerous sources of infection for embryos show how well PCV2 adapted in the pigs, and it does not end here, as PCV2 belongs to the viruses of the “slow and low” tactic [14]. PCV2 replication is inefficient as the melting pot rolling circle replication used for PCV2 propagation, as it is a slow form of the rolling circle replication and, after one whole genome is replicated, the process is terminated [6]. Poor replication for PCV2 was also found in vitro [15]. Moreover, the simultaneous presence of both PCV2 genotype group members, PCV2a and PCV2b, for PCV2 replication [7] complicates the virus replication and thus its propagation. However, this slow modus of replication aids PCV2 to stay hidden and inconspicuous in embryos and fetuses that need a lot of energy for development, which otherwise could be easily overwhelmed by the virus replication and resulting energy demand. This slow replication modus also guarantees minimal damage to the tissue and minimal visibility to the immune system as described for latent viruses [14]. 

Interestingly, all of the lymph nodes in close proximity to the ovaries or testicles were highly populated with latent PCV2 infected cells (Table 1, Figure 2). These infected cells of the lymphatic system have not been specifically identified thus far. There were numerous cell types possibly displaying one or another characteristic of these PCV2 latent infected cells that were visualized for the first time in Klausmann et al. [13] by FISH analysis, and they were also thought to be of the dendritic cell type. It could also be that they are of the innate lymphoid cell type (ILC), which was described by Barrow et al. [44]. ILCs’ imprinting functions in embryonic development as well as its innate connection with the adaptive immune system [44] make them a prime target of investigations.

Furthermore, the current report also investigated the thymi of stillborn fetuses carrying PCV2 latent infected cells (Table 1). We think that the presence of PCV2 latent infected cells in the thymus leads to the depletion of PCV2 specific T cell clones [13], as noted by other research scientists [27]. We believe this is the driving force of our present observations and it is an indication of virus interference in the host’s central tolerance. We found that since the genetic shift from PCV2a to PCV2b genotype group members in 2003 [45], the dominant sequences that we identified from embryos, fetuses, stillborn fetuses, weaners, or adult pigs were of the PCV2b genotype. For the quick identification of the PCV2 genotype, we used the original PCR, which was described for the first time in 2009 [45]. Even though the analyzed sequence of 180 base pairs was comparatively small, it covered the signature sequence of PCV2 capsid protein [46] and, therefore, was of major help for rough genotype characterization. In the PCR amplificate, it was difficult to differentiate the PCV2b genotype group members as one critical nucleotide within the flanking oligonucleotide sequences is presented as wobble nucleotide “Y”. Therefore, for a more intricate analysis, we also amplified the whole ORF2 of PCV2 from pigs that were in vivo transfected with a cocktail of equal amounts of PCV2a and PCV2b/d genotype group members. In the interpretation of the outcome of this experiment, one has also to consider our data from Khaiseb et al. [7], where we found a dominant genotype group member and, in the background, there were other PCV2 genotype group members in the same pigs and in the same cell. Moreover, one needs to also take into account that the negative selection in the thymus is not 100% of T cells, which recognize self [29]. The whole ORF2 amplificate of the two controls and one CysA weaner indicated that the PCV2b genotype group member, JX512857, was the dominant PCV2 virus in the experimental weaner cohorts. Thus, it must have already influenced central tolerance in the experimental cohorts. As part of the PCV2b genotype group member and the 100% nucleotide identity with PCV2b-CH [45], these nucleotides were quite familiar to us from the Swiss pig population [7,24,31]. Consequently, one genotype group member, JX512857, was found in 15 out of 17 *in vivo* transfected weaners—independent of cyclosporine A treatment—in spite of competition with the other eight PCV2 genotype group members. Two samples out of nine with mixed group member genotyping were from the pig cohort of the IVI (Mittelhäusern, Bern), which had developed independently to our weaner cohort. It may well be that the genetic PCV2 composition of these weaners was more fluent, and that the dominant genotype group member was not yet completely settled. A similar scenario with the co-existence of three different genotypes (PCV2a, PCV2b, and PCV2d) was recently published from the Yunnan province [47], and it would be interesting to test these weaner populations with our hypothesis and determine which of the PCV2 genotype group member(s) would dominate after in vivo transfection with mixed recombinant PCV2 genotype group members.

It is also important to note that both co-injected PCV2b genotype group members, JX512858 and JX512860, are only in one nucleotide, namely a cytosine, at position 270 of the ORF2, which is different from JX512857, which codes for a thymine. However, this is a silent mutation. Nevertheless, these three PCV2b group members differ from each other in at least one amino acid of the Rep protein. This small difference seems to have given the advantage to JX512857, as with its antigenic dominance, a few more T-cell clones were depleted during T cell development in regard to the other PCV2b group members leading to the viral propagation advantage of JX512857. From the mixed PCV2 genotype group member injection experiments, it seems that one PCV2d genotype group member, JX512856, was not inherently more pathogenic as other research scientists have claimed [11]. It also appears that the PCV2 genotype group member dominance during early pig development and the resulting interference with the central tolerance of the host is the decisive reason why a PCV2 genotype group member seems to appear more pathogenic.

This conclusion is further supported by another pig transfection experiment, where we transfected weaners with the background of the PCV2b genotype group members either with a cocktail mix of recombinant PCV2a genotype group members, or separately with a cocktail mix of recombinant PCV2b/d genotype group members. The immune system of the eight involved weaners recognized the injected viruses by the appearance of anti-PCV2 IgM antibodies (Table 2 and Table 3). Still, the immune system mounted an efficient response against the PCV2a genotype group members and, thus, we uncovered in only one weaner, one time, a PCV2 viremia over 10^5^ templates/mL blood (Table 2 and Table 3). This PCV2 viremia is normally classified as a subclinical infection and it is quite common in pigs [48]. Vice versa, this reaction of the host immune responses indicated a lack of a strong adaptive immune response against the PCV2b/d genotype group members, as in all PCV2b/d-weaners, a viremia with template levels higher than 10^6^ templates/mL blood were discovered, and this viremia lingered (Table 2 and Table 3). These PCV2b/d-weaners’ viremia was similar to the previously described PCV2ab/d-weaners’ viremia with all three transfected genotype group members and without cyclosporine A treatment in Klausmann et al. [13]. 

These current findings support the idea that the distortion of the central tolerance is the major pathogenicity mechanism of PCV2 and not that PCV2 genotype group members on their own have an intrinsic, genetically based, different pathogenicity.

The idea of circovirus involvement in central tolerance is pretty new [13], and these detailed data are unique for viruses in general; hence, it is difficult to find parallels within the single-stranded DNA viruses (e.g., *Circoviridea* or *Anneloviridae*). Furthermore, research scientists have not specifically focused on designing experiments around questions that have since risen around PCV2 central tolerance engagement. This may change in the foreseeable future, as some key parallels to PCV2 pathogenicity are also obvious when compared with better studied viruses, such as the chicken infectious anemia virus (CIAV) of the *Anneloviridae* family and gyrovirus genera [2]. CIAV causes chicken anemia disease in young chicks, is lymphotropic, and causes accelerated involution of the thymus that lead to the reduction of peripheral T cells [49,50], which are also hallmarks for PCVD [13,48]. Moreover, CIAV also influences T cell receptor signaling in the thymus [50], which was also seen for PCV2 [13]. “Latent” CIAV can be transmitted vertically over generations without consequences for the birds or the infected embryos [51]. CIAV latent infections were noted after sudden seroconversion of sexual mature birds within their first laying cycle [52]. Through the male’s reproductive organs, CIAV infections can occur through the ejaculate [52]. These are a few selected similarities between the two viruses in regard to respective host vertical transmission and disease. However, even fewer data are available regarding human diseases of gyroviruses or cycloviruses, another family member of the *Circoviridae*. Only time will tell how much impact this presented work, and our previous engagement of elucidating PCV2 involvement in the maturation of T cells and, more specifically, in host central tolerance, will have for veterinary and human medicine.

## 4. Materials and Methods

### 4.1. Ethics Statement

The animal experiments and protocols that were followed in the present study were approved by the Animal Welfare Committee of the Canton Bern (authorization NO 98/09 and license BE26/11) and by the Animal Welfare Committee of Canton Zurich (authorization no. 227/2010), respectively. The handling of and experiments with pigs were carried out in accordance with the European Union and Swiss standards (Swiss Animal Welfare Act “Tierschutzgesetz SR455”).

### 4.2. Animals and Weaner Transfection Experiments

All pigs and pig fetuses were from well-managed Swiss breeding farms with no clinical signs of disease. This includes no PCVD symptoms or any history of PCVD, nor any association with major respiratory or intestinal pathogens. They were genetically of the origin of Swiss White Large (SWL) or Landrace (LR), or a crossbred of the two.

We present additional data from the pig infection experiments at the Institute of Virology and Immunology (IVI, Mittelhäusern, Switzerland) and the Institute of Veterinary Pathology (Zurich, Switzerland), which were completed from 2011–2012 [13]. From these weaners in vivo transfection experiments, we collected whole blood samples from (i) weaners controls with mock in vivo transfection, (ii) cyclosporine A alone treated weaners (CysA) with mock in vivo transfection, (iii) from in vivo transfected weaners with all three genotype group members with or without cyclosporine A. 20 blood samples of these 18 weaners were used for complete ORF2 sequencing. Concomitantly, in the IVI, the blood samples were taken from the eight weaners transfected with PCV2a or PCV2b/d genotype group members separately. The whole blood samples were stored in ethylenediaminetetraacetic acid (EDTA) coated tubes S-Monovette (Sarstedt, Sevelen, Switzerland) at −20 °C.

### 4.3. Handling of Fertilization and Embryo Collection

Three sows were chosen for embryo collection, as they would have been slaughtered due to their age. These three sows were artificially inseminated twice during estrus after weaning their piglets. The sows were slaughtered and their uterus was removed three days after the second insemination. The uterine horns were ligated at the tip of the horn at about 8 cm distal from the oviduct junction. A small hole was punctured in the middle of the oviduct. Through this hole, the fertilized embryos were collected by rinsing the ligated horn tips and then placed into petri dishes where the embryos were washed three times in order to remove loosely attached PCV2.

### 4.4. Template Quantification by Real-Time PCR from Whole Blood

We collected pig whole blood from the jugular vein and directly transferred it into EDTA coated tubes S-Monovette (Sarstedt, Sevelen, Switzerland). We used an in-house SYBR-Green based real-time PCR, as described in Klausmann et al. [13]. The real-time PCR was optimized and adjusted on a dilution series of plasmid containing PCV2 ORF2 fragments, as described in Wiederkehr et al. [45].

All of the PCR amplificates were sequenced from Microsynth AG (Balgach, Switzerland).

### 4.5. Recombinant PCV2 Genotype Group Member-DNA Preparation for PCR Optimization and In Vivo Transfection by JetPei

For polymerase chain reaction (PCR)-optimization and in vivo transfection by JetPei-carrier (Polyplus transfection, Illkirch, France) [13], we first needed to liberate whole PCV2 genotype group members from each hybrid plasmid, pCR2.1-TOPO (pCR2.1-TOPO, Invitrogen, Basel, Switzerland) by EcoR I (New England Biolabs, Bioconcept, Allschwil, Switzerland) digestion and purification over a size exclusion column (MinElute PCR Purification column, Qiagen; Hombrechtikon, Switzerland). As intramolecular reactions are faster than intermolecular reactions, we directly ligated (T4-DNA Ligase; New England Biolabs, Allschwil) the DNA mix from the plasmid backbone and whole PCV2 group member digestion/purification. This ligation was again purified over a column (MinElute PCR Purification column, Qiagen; Hombrechtikon, Switzerland). The resulting whole PCV2 group member DNA ligation mix was used as a template for optimizing the PCR reaction conditions and in vivo transfection experiments, as described in Klausmann et al. [13].

### 4.6. In Vivo Transfection by JetPei Carrier

In vivo transfection was previously described in Klausmann et al. [13]. In short, each weaner was transfected in vivo with a total of 20 μg PCV2 recombinant DNA that was prepared by the restriction enzyme digest and ligation as described above. Half of this 20 μg recombinant plasmid mix was taken up in JetPei (Polyplus transfection, Illkirch, France) and the other half in JetPei-Man carrier (Polyplus transfection, Illkirch, France). Both of the transfection mixes were gently mixed together and again separated in half volumes to inject both in weaner’s neck region. Equal DNA amounts of the PCV2a genotype group members (6.7 μg each), JX512853, JX512854, and Stoon10-10, or the PCV2b/d genotype group members (3.3 μg each), JX512855, JX512856, JX512857, JX512858, JX512859, and JX512860 were separately injected in weaners.

### 4.7. Amplification of Whole PCV2 ORF2 and Classification of Embryonic Infections

We used the previously described method in Wiederkehr et al. to define genotype group members from paraffin-embedded formalin fixed sexual organ blocks [45]. Paraffin slices were deparaffinized with xylene-ethanol extraction, and the remaining pellets were digested with 20 µL proteinase K (20 mg/mL proteinase K, Roche Diagnostics GmbH, Mannheim, Germany).

We extracted DNA for PCR reactions from whole blood or paraffin-embedded tissue slices using the DNeasy^®^ Blood & Tissue Kit from Qiagen (Hombrechtikon, Switzerland). All PCR reactions, from the cloning of whole PCV2 ORF2, to the virus classification of embryonic infections were optimized on dilution series of whole PCV2 recombinant plasmid mix with corresponding polymerase and oligonucleotide pairs, similar to the description of PCV2-plasmid in Wiederkehr et al. [45]. Even though we optimized PCR reactions, we were only able to amplify with proof-reading polymerase sequences from samples that contained at least a few hundred PCV2 templates per µL DNA eluate.

We amplified PCV2 ORF2 from whole blood in order to determine the dominant PCV2 genotype group member from in vivo tranfected weaners. This DNA was extracted from the experimental pigs [13] and was amplified using the oligonucleotide pair: (A) 5′ GGA GGA TTA CTT CCT TGG TAT TTT GG 3′ and (B) 5′ GGT GCT GCC GAG GTG CTG 3′ and the proof-reading Vent^®^ DNA polymerase (New England Biolabs, Bioconcept, Allschwil). We added Vent^®^ DNA polymerase (New England Biolabs, Bioconcept, Allschwil, Switzerland) to the PCR reaction mix with the extracted DNA and oligonucleotide pair at 80 °C to mimic a hot start at the beginning of the PCR reaction. This was followed by a step of 30 s at 94 °C with ensuing normal PCR amplification reaction.

For both DNA extracted from paraffin-embedded tissue slices and from three-day-old embryos directly, we used nested PCR in order to investigate the presence of PCV2 DNA. For the first amplification of the oligonucleotide pair, (A) and (B) were used. For the second amplification, we used oligonucleotide pairs, as described in Wiederkehr et al. [45]. The PCR amplification was performed with the help of the PhusionFlash (Thermo Fisher Scientific, Reinach, Switzerland) polymerase, which has proofreading activities.

### 4.8. Identification and Location of PCV2 Infections in Female and Male Genital Tracts of Stillborn Fetuses by FISH

PCV2 infection analysis of stillborn fetuses was done by FISH analysis with fluorescence microscopy and confocal laser scanning microscope (CLSM). The pig fetuses were from six farms without porcine circovirus diseases or any PCV2 associated diseases. Their genital organs and thymi were investigated by necropsy, immunohistochemistry [45], and FISH analysis [7] for the presence of PCV2. The FISH analysis sections were analyzed with Hamamatsu’s NanoZoomer 2.0 HT with a fluorescence option. We also used a Leica fluorescence microscope and CLSM to confirm and refine the analysis. To identify PCV2 infected cells in stillborn fetuses’ genital tracts and other organs, we used a triple staining technique consisting of DAPI to visualize the cell nucleus, the 5′ and 3′ Atto-565 labeled AB-oligonucleotide that recognizes the ORF2 (*cap*) [7], and the 5′ and 3′ Dyomics-630 labeled P2O-O1r-oligonucleotide recognizes the sequence overlapping with ORF1 (*rep*) [13]. The stainings were performed with help of the Discovery instrument (Ventana Medical Systems from Roche AG, Oro Valley, AZ, USA).

### 4.9. Anti-PCV2-IgG and IgM ELISAs

We used the competitive ELISA, SERELISA^®^ PCV2 Ab Mono Blocking Systems of Synbiotics Corporation Europe SAS (Lyon, France) and the INGEZIM CIRCOVIRUS IgG/IgM^®^ of Ingenasa (Madrid, Spain) assay in order to detect anti-PCV2-IgG and anti-PCV2-IgM in the pigs’ whole blood. The assays were used according to the instructions and Kurmann et al., 2011 [21]. Only anti-PCV2 IgG concentration above subclinical infection levels (with ≤900 ELISA units (EU)) were counted as IgG-positive weaners if they occurred once in 10 consecutive blood sampling attempts during the 51 days p.t. [13].

## Figures and Tables

**Table 1:** Stillborn fetuses’ reproductive tracts associated PCV2 cell Infections.

Stillborn Fetus Nr.	Sex	Farms	Sequence	Thymus	Lymph Node	Testicles	Epidi-dymis	Sexual Glands	Ovaries	Oviduct	Uterus Horn
**17-0112**	f	farm 1	PCV2b	++++++-	+++				+	-	-
**17-0113**	m	farm 1	PCV2b	+++	no	+	-	-			
**17-0114**	m	farm 1	PCV2b		no	+	-	-			
**17-0115**	m	farm 1	PCV2b		no	+++	++	+			
**17-0116**	f	farm 1	PCV2b		no				-	-	-
**17-0117**	m	farm 1	No PCV2 similarity	++++++-	+++	++	0	+			
**17-0119**	m	farm 1	No PCV2 similarity	+++++	++	+	-	-			
**17-0120**	f	farm 1	No PCV2 similarity	++++++	++				+	0	0
**17-0121**	f	farm 1	No PCV2 similarity		++				-	-	-
**17-0122**	f	farm 1	No PCV2 similarity		no				+	0	0
**17-0123**	f	farm 1	No PCV2 similarity	+++++-	+++				-	-	-
**17-0124**	m	farm 1	No PCV2 similarity		no	+	-	+			
**17-0125**	m	farm 1	No PCV2 similarity	+++++	+++	+	0	-			
**17-0126**	f	farm 1	PCV2b		no				0	0	0
**17-0127**	m	farm 1	No PCR Product	+++++	no	+	0	0			
**17-0128**	f	farm 1	PCV2b		+++				-	0	0
**17-0129**	f	farm 1	No PCV2 similarity	++	++				-	0	0
**17-0156**	m	farm 1	No PCV2 similarity		+++	+	-	-			
**17-0223**	f	farm 1	No PCV2 similarity		++				-	-	-
**17-0224**	m	farm 1	No PCV2 similarity		no	++	0	no			
**17-0229**	f	farm 1	PCV2b		no				-	-	-
**17-0231**	m	farm 1	No PCV2 similarity		no	-	-	no			
**17-0234**	f	farm 1	No PCV2 similarity	+	++				0	-	0
**17-0235**	f	farm 1	PCV2b	+++++	no				-	0	-
**17-0236**	f	farm 1	No PCV2 similarity		no				+	-	0
**17-0237**	m	farm 1	No PCV2 similarity		no	+	0	no			
**17-0238**	m	farm 1	No PCV2 similarity		++	+	0	no			
**17-0239**	f	farm 1	PCV2b		no				0	-	0
**17-0245**	m	farm 1	No PCV2 similarity		no	no	0	no			
**17-0311**	m	farm 1	No PCV2 similarity		no	+	0	+			
**17-705**	m	farm 2	No PCR Product	+	no	no	0	no			
**17-706**	m	farm 2	No PCR Product	+++++	no	no	0	no			
**17-707**	f	farm 2	No PCR Product	+	+++				-	0	-
**17-708**	f	farm 2	PCV2b	-	no				0	-	0
**17-709**	m	farm 2	No PCR Product	++++	no	no	0	no			
**17-0719**	f	farm 3	No PCR Product		no				-	0	-
**17-0720**	f	farm 3	No PCR Product	+++	no				+	0	0
**17-0721**	m	farm 3	No PCV2 similarity		no	no	0	no			
**17-0722**	m	farm 3	No PCR Product	++++++	++	0	0	0			
**17-0723**	f	farm 4	No PCR Product	-	+++				-	0	0
**17-0724**	m	farm 4	No PCR Product	++++++	+	+++	0	++			
**17-0725**	m	farm 4	No PCR Product	++	no	+	0	no			
**17-0726**	m	farm 4	No PCR Product	++++++-	no	+	-	0			
**17-0736**	f	farm 5	No PCR Product	++++	no				0	-	0
**17-0737**	m	farm 5	PCV2b	+-	no	no	0	-			
**17-0738**	m	farm 5	PCV2b	+	no	+	+	no			
**17-0739**	f	farm 5	No PCV2 similarity	++	no				0	0	0
**17-0740**	m	farm 5	No PCR Product	+-	no	+	0	-			
**17-0741**	m	farm 5	No PCV2 similarity		no	no	0	-			
**17-0742**	f	farm 6	No PCR Product	+-	+++				-	0	0
**17-0743**	m	farm 6	No PCR Product	+++++	no	+	0	no			
**17-0744**	m	farm 6	No PCV2 similarity	++++	no	no	0	no			
**17-0745**	m	farm 6	No PCV2 similarity	++++	no	no	0	no			
**17-0746**	f	farm 6	No PCV2 similarity	++	no				-	0	0
**17-0747**	m	farm 6	No PCV2 similarity	++++	no	+	0	no			

Legend for sexual organs: no: no organ present; 1–4 PCV2 infected cells: –; 5–20 PCV2 infected cells: +; 21–40 PCV2 infected cells: ++; >40 PCV2 infected cells: +++. Legend for Thymus: Indicates few PCV2 cell infections to many infected cells from: ‘-’ to ‘++++++-’. Legend for Lymph node: Indicates few PCV2 cell infections to many infected cells ‘+’ to ‘+++’ no: no lymph node present. These are estimates per tissue section and ”–“ indicates the half estimate of “+” if not otherwise indicated.

**Table 2:** Weaners PCV2 genotype group members’ transfections.

Weaner ID No.	Sex	PCV2 Transfected Groups	Anti-PCV2 IgM		Virusload		Anti-PCV2 IgG	IHC of Tonsil Tissue	PCVD
			Days p.t.	51d p.t.	Highest Conc. Days p.t. (Virus Concentration Cutoff < 10^5^ = 0)	51d p.t.	Value ≤ 900 EU	Centrofollicular PCV2 Antigen Staining	
S11-0933	m	control	neg	neg	0	0	neg	0	0
S12-1570	m	control	neg	neg	0	0	neg	0	0
S11-0935	f	control CysA	neg	neg	0	0	neg	0	0
S11-0936	f	control CysA	neg	neg	0	0	neg	0	0
S11-0934	m	control	neg	neg	0	0	neg	0	0
S12-1566	m	control CysA	neg	neg	0	0	neg	0	0
S11-0925	m	PCV2b/d	35d	neg	35d: 7 × 10^7^	0	neg	0	0
S11-0926	m	PCV2b/d	20d, 26d, 35d, 41d	neg	16d: 3 × 10^7^	1 × 10^6^	neg	0	0
S11-0927	m	PCV2b/d	26d, 35d, 41d	pos	20d: 6 × 10^7^	4 × 10^6^	neg	13	0
S11-0928	m	PCV2b/d	26d, 35d, 41d	pos	26d: 4 × 10^6^	3 × 10^5^	neg	0	0
S11-0929	m	PCV2a	26d,35d, 41d	neg	0	0	neg	0	0
S11-0930	m	PCV2a	35d, 41d	neg	0	0	neg	0	0
S11-0931	m	PCV2a	35d, 41d	neg	0	0	neg	0	0
S11-0932	m	PCV2a	35d, 41d	neg	35d: 2 × 10^5^ (only once)	0	neg	0	0

**Table 3:** Summary of transfections and analyzed parameters.

In Vivo Transfection	IgM	IgM 51d *	IgG	Virus +	Virus #	Virus 51d *,+	IHC Tonsils ⊥	PCVD
PCV2a	4/4	0/4	0/4	0/4	0/4	0/4	0/4	0/4
PCV2b/d	4/4	2/4	0/4	4/4	3/4	2/4	1/4	0/4
control (mock)	0/6	0/6	0/6	0/6	0/6	0/6	0/6	0/6

* days of slaughter and necropsy post-transfection. + virus > 10^6^ genomes/mL (blood). # virus > 10^7^ genomes/mL (blood). ⊥ centrofollicular and more than 3 cells stained immunohistochemically.

## Supplementary Materials

The following are available online at https://www.mdpi.com/2076-0817/9/10/839/s1, Table S1: Sequences.
